# Association of Anaesthetists guidelines: the use of blood components and their alternatives

**DOI:** 10.1111/anae.16542

**Published:** 2025-01-09

**Authors:** Akshay Shah, Andrew A. Klein, Seema Agarwal, Andrew Lindley, Aamer Ahmed, Kerry Dowling, Emma Jackson, Sumit Das, Divya Raviraj, Rachel Collis, Anna Sharrock, Simon J. Stanworth, Paul Moor

**Affiliations:** ^1^ Nuffield Department of Clinical Neurosciences and NIHR Blood and Transplant Research Unit in Data Driven Transfusion Practice University of Oxford Oxford UK; ^2^ Department of Anaesthesia, Hammersmith Hospital Imperial College Healthcare NHS Trust London UK; ^3^ Department of Anaesthesia and Intensive Care Royal Papworth Hospital Cambridge UK and Chair, Working Party, Association of Anaesthetists; ^4^ Department of Anaesthesia, Manchester University NHS Foundation Trust Manchester UK and the Association of Anaesthetists; ^5^ Department of Anaesthesia Leeds Teaching Hospitals NHS Trust and Royal College of Anaesthetists; ^6^ Department of Cardiovascular Sciences University of Leicester Leicester UK; ^7^ Department of Anaesthesia and Critical Care, Glenfield Hospital University Hospitals of Leicester NHS Trust Leicester UK and the Association for Cardiothoracic Anaesthesia and Critical Care (ACTACC); ^8^ Transfusion Laboratories Southampton University Hospitals NHS Foundation Trust; ^9^ Department of Cardiothoracic Anaesthesia, Critical Care, Anaesthesia and ECMO, Wythenshawe Hospital Manchester University NHS Foundation Trust Manchester UK and Intensive Care Society UK; ^10^ Nuffield Department of Anaesthesia Oxford University Hospitals NHS Foundation Trust Oxford UK and the Association of Paediatric Anaesthetists of Great Britain and Ireland and the Royal College of Anaesthetists; ^11^ Resident Doctors Committee, the Association of Anaesthetists; ^12^ Department of Anaesthesia University Hospital of Wales Cardiff UK and the Obstetric Anaesthetists Association; ^13^ Department of Vascular Surgery Frimley Health NHS Foundation Trust Frimley UK; ^14^ NIHR Blood and Transplant Research Unit in Data Driven Transfusion Practice, Radcliffe Department of Medicine University of Oxford and on behalf of the British Society of Haematology and NHS Blood and Transplant; ^15^ Department of Anaesthesia Derriford Hospital Plymouth UK and the Defence Anaesthesia Representative

**Keywords:** bleeding, major haemorrhage, peri‐operative, tranexamic acid, transfusion

## Abstract

**Background:**

The administration of blood components and their alternatives can be lifesaving. Anaemia, bleeding and transfusion are all associated with poor peri‐operative outcomes. Considerable changes in the approaches to optimal use of blood components and their alternatives, driven by the findings of large randomised controlled trials and improved haemovigilance, have become apparent over the past decade. The aim of these updated guidelines is to provide an evidence‐based set of recommendations so that anaesthetists and peri‐operative physicians might provide high‐quality care.

**Methods:**

An expert multidisciplinary, multi‐society working party conducted targeted literature reviews, followed by a three‐round Delphi process to produce these guidelines.

**Results:**

We agreed on 12 key recommendations. Overall, these highlight the importance of organisational factors for safe transfusion and timely provision of blood components; the need for protocols that are targeted to different clinical contexts of major bleeding; and strategies to avoid the need for transfusion, minimise bleeding and manage anticoagulant therapy.

**Conclusions:**

All anaesthetists involved in the care of patients at risk of major bleeding and peri‐operative transfusion should be aware of the treatment options and approaches that are available to them. These contemporary guidelines aim to provide recommendations across a range of clinical situations.

## Recommendations

Pre‐operative:

1. All patients should have their haemoglobin concentration measured before listing for major elective surgery.
2. Where blood transfusion is anticipated, this and alternatives to transfusion should be discussed with the patient before surgery, and their consent should be documented according to local protocols.

Minimising peri‐operative blood loss:

3 Management of anticoagulants in the peri‐operative period should balance the risk of bleeding with the risk of thrombosis.
4 The use of cell salvage and antifibrinolytics, such as tranexamic acid, is recommended in all patients who are bleeding, where blood loss > 500 ml (> 8 ml.kg^‐1^ in children weighing > 10 kg) is possible and/or in patients are unable to receive donor blood.
5 Minimise iatrogenic anaemia by managing blood sampling appropriately.

Recognition and management of major bleeding:

1. Early recognition of obstetric bleeding is essential and should be measured accumulatively, leading to a clear escalation plan of intervention and involvement of the multidisciplinary team.
2. Every institution should have a major haemorrhage protocol which is regularly audited and reviewed. They should be concise but targeted, and allow for immediate release and protocolised administration of blood components.
3. In an emergency, surgical implementation of damage/proximal control strategies may be required until haemorrhage control is achieved.
4. Group O red blood cells (RBCs) for transfusion should be readily available in the clinical area, in case haemorrhage is life‐threatening. Group‐specific RBCs should be made available by the laboratory as soon as possible after receiving correctly labelled samples and being informed of the emergency requirement for blood, and according to local laboratory protocols.
5. During major haemorrhage due to trauma, preference should be given to transfusing RBCs and fresh frozen plasma (FFP). Clear fluids (crystalloids) should be avoided unless there is profound hypotension and no imminent availability of blood components.
6. Blood components should be prescribed for children as a volume (ml.kg^‐1^) rather than in units.
7. Patients who continue to bleed actively should be monitored by point‐of‐care and/or regular laboratory tests for coagulation, fibrinogen and platelet counts and/or function. A guide for transfusion should be: FFP if INR > 1.5; cryoprecipitate if fibrinogen < 1.5 g.l^‐1^ (< 2.0 g.l^‐1^ in obstetrics); and platelets if platelet count < 50 × 10^9^.l^‐1^ (< 75 × 10^9^.l^‐1^ in obstetrics).

## What other guidelines currently exist?

Other guidelines are available, many of which are quite recent, but none cover the breadth and scope of UK anaesthesia practice (online Supporting Information Appendix S1).

## Why were these guidelines developed?

Transfusion medicine is a rapidly evolving field with several practice‐defining trials published in the last few years, along with constantly improving haemovigilance systems. Delayed recognition of bleeding and delayed transfusion are both associated with adverse outcomes. As a result, there is a need for a relevant, contemporary clinical guideline for practising UK anaesthetists, critical care staff, allied health professionals and those from other relevant specialties and backgrounds.

## How and why does this statement differ from existing guidelines?

This is an updated guideline, based on data from recent studies, and includes new sections on pre‐hospital management and acute gastrointestinal bleeding. This guideline advocates strongly for a multidisciplinary approach involving close working between anaesthetists, intensivists surgeons, obstetricians, haematologists, blood bank staff and other relevant departments to allow for safe, timely and targeted transfusion support.

## Introduction

Transfusion medicine is a constantly evolving field, encompassing not the just the use of blood components but also consideration of alternative agents, novel cellular therapies and maintaining donor health. Anaesthetists are often involved in decisions to administer blood components and/or alternatives, as well as the management of major haemorrhage. The evidence base continues to expand with an increasing number of randomised controlled trials (RCT) across multiple clinical settings. There is also now a well‐established interest in improving patient safety during transfusion through national haemovigilance systems such as Serious Hazards of Transfusion (SHOT) [1].

Allogeneic RBC transfusion can be lifesaving, but this is a scarce and costly resource as highlighted by recent national blood shortages and changes in donor patterns during the COVID‐19 pandemic [2]. Around 1.5 million RBC units are issued by blood services in the UK per year [3]. Approximately one‐third of these are for patients undergoing major surgery, in particular, cardiac surgery, vascular surgery and cancer surgery [4]. Use of blood components also remains high in major trauma, intensive care and obstetrics.

To ensure appropriate use of blood components and improve patient outcomes, the concept of patient blood management (PBM) was endorsed as a standard of care in the UK more than 10 years ago. In brief, PBM [5, 6] is the timely application of evidenced‐based principles focusing on three ‘pillars’: detection and management of pre‐operative anaemia; minimising blood loss and treating coagulopathy; and harnessing and optimising the patient's physiological reserve in the setting of anaemia. It should also take an individualised approach, with consideration of the clinical setting and patient preference (where possible). Serial national comparative audits and practice surveys have demonstrated improvements in uptake of PBM but significant gaps and variation in practice remain [7, 8]. The aim of this guidance is to update previous guidance about the appropriate use of blood components and their alternatives, that neither compromise the recipient nor expose them to unnecessary risks. Strategies to minimise blood loss and improving the process of transfusion will also be discussed. Treating peri‐operative anaemia and use of cell salvage are key components of PBM and readers are referred to specific guidance on these topics [9, 10].

## Methods

We convened a consensus panel comprising 11 members with expertise in anaesthesia, patient blood management, haematology, obstetrics, cardiac and non‐cardiac surgery, major trauma and critical care. This panel also represented different professional societies.

The consensus process incorporated a three‐step Delphi method, which took place between January 2022 and January 2023, to formulate a set of recommendations. All Working Party members independently rated the importance of each recommendation using a 4‐point Likert scale using an online survey tool (http://www.surveymonkey.co.uk). Members were able to suggest changes to the wording of recommendations to improve the clarity and the content and suggest possible mergers. Following the final round of scoring, and after discussion, a final list of 12 recommendations was produced.

Searches for relevant publications were carried out on MEDLINE, EMBASE, CINAHL, CENTRAL and the Transfusion Evidence Library including the following example search terms: – blood; transfusion; haemorrhage; bleeding; and peri‐operative. References known to the Working Party members and other guidelines published by national and international organisations were consulted. The Working Party focused on systematic reviews, meta‐analyses and RCTs to generate recommendations but acknowledged that recommendations on laboratory and organisational aspects would largely be based on observational studies. Where evidence was limited, the Working Party sought to provide pragmatic guidance.

## Results

### Process of transfusion

Positive patient identification is paramount prior to any blood transfusion episode, to minimise wrong‐blood‐in‐tube events and risks of ABO incompatibility [11]. All patients receiving a blood transfusion must wear a patient identification band. The minimum patient identifiers provided on the transfusion request form and sample include surname, forename, date of birth, and the hospital unique identification number, or NHS number (or equivalent). In situations where the patient's identity may be unknown, an alternative identification system must be in place. Follow local guidelines but, as an example, at least one unique identifier must be used (e.g. a randomly generated seven‐digit number with a prefix), a naming convention (suggest randomly generated from an edited phonetic alphabet), a date‐of‐birth system and the patient's sex. Once the patient's identity is known, a new identification band must be attached to the patient, and a new transfusion sample must be collected and labelled with the patient's details.

Blood samples must be collected and hand or electronically‐labelled at the patient's side by appropriately trained personnel. Two samples are not always needed if the patient has a suitable ‘historical’ sample on file. Historical samples may be available in a format that makes them useful for information to use in conjunction with the current (valid) sample [12]. Where patient identification is sufficient to assure that the historical sample is from the same patient as the current one, the historical sample may be valid as the ‘group‐check’ sample to allow issue of ABO compatible RBCs to meet the two‐sample recommendation. However, to meet the criteria for electronic issue of RBCs against the current sample, the historical group must have the same patient identification and be transmitted electronically (with no manual intervention). If neither of these are present, then two samples will be required.

If a suitable historical sample is available, only one group and screen sample is required which should be taken according to national guidelines [12]. In brief, if a patient has not previously received a transfusion, is not pregnant or has not been pregnant in the last 3 months, the sample is valid for 7 days, but we recognise that some hospital use 72 h. Separating and storing the plasma at −30°C can increase the sample validity to 3 months. If a patient has received a transfusion or is/has been pregnant in the last 3 months, the sample must be less than 3 days old at the beginning of any transfusion. Therefore, in patients scheduled to undergo surgery at risk of major blood loss, it is important to ascertain whether they may have received a transfusion at any other hospital within a three‐month period. If a patient has received a blood transfusion or been pregnant within the previous 3 months, then the sample is only valid for 72 h, which is from the time the sample is taken to the subsequent transfusion.

Requests for blood components may be written, electronic or done by telephone. Ensure the request is clearly communicated to the laboratory including volume, product required, special requirements, location and date/time required. Prepared blood components may be stored within the laboratory, held in a satellite blood fridge close to the clinical location or distributed via a remote issue system. The method of storage used will depend on local hospital blood transfusion policy. Selection of RBCs will proceed through either electronic issue, serological crossmatch or emergency issue. Serological crossmatch can detect ABO and non‐ABO red cell antibody incompatibility between donor cells and recipient plasma and is the default technique when electronic issues is contra‐indicated or in the absence of a functioning, validating information technology system. Detailed guidance on pre‐transfusion compatibility procedures and electronic systems in transfusion laboratories is available elsewhere [12, 13].

During the transfusion episode, patient monitoring is essential to identify and manage adverse reactions. Dyspnoea and tachypnoea are typical early symptoms of serious transfusion reactions; therefore, the respiratory rate should be monitored throughout transfusion as recommended by the National Institute for Health and Care Excellence (NICE) [14]. Other observations including pulse, blood pressure and temperature should be undertaken and documented for each unit transfused. As a minimum, observations should be completed and recorded before the start of the transfusion (within 60 min), 15 min after the start of each unit and within 60 min of the end of transfusion [11].

Transfusion‐associated circulatory overload (TACO) is now the most common cause of transfusion‐related mortality and major morbidity [1]. It is broadly defined as acute or worsening respiratory compromise and/or acute or worsening pulmonary oedema during or up to 12 h after transfusion, cardiovascular changes (tachycardia, hypertension) not explained by the patient's underlying condition, evidence of fluid overload and supportive result of a relevant biomarker (e.g. brain natriuretic peptide) [1]. Risk factors for developing TACO include older, non‐bleeding patients (age > 70 years), comorbidities (heart failure, renal failure and hypoalbuminaemia), low body weight and rapid transfusion. Peri‐operative considerations in such patients include assessing the need for transfusion, body weight dosing of RBCs, slow transfusion, close monitoring of vital signs and fluid balance and prophylactic diuretic prescribing. Various infographics and TACO checklists are available from SHOT [1].

The incidence of febrile, allergic and hypotensive reactions which occur within 24 h following transfusion, and for which no other cause is found, is increasing [1, 15, 16]. This may, in part, be due to increased reporting. Red blood cell units are usually associated with febrile‐type reactions, whereas plasma and platelets more commonly cause allergic reactions. Current recommendations from SHOT advise to not use steroids and/or antihistamines indiscriminately [1]. Repeated doses of steroids may further suppress immunity in patients who are immunocompromised. Instead, a more personalised approach is recommended, which is tailored to the patient's symptoms and signs to distinguish between febrile and allergic reactions. For febrile reactions, only intravenous paracetamol may be required. For allergic reactions, only an antihistamine should be administered. If a severe reaction and/or anaphylaxis is suspected, local anaphylaxis protocols should be followed.

Intra‐hospital transfer of patients may occur during transfusion. Large numbers of units of RBCs should not be transferred with the patient and the receiving clinicians should ensure blood is returned to appropriate storage and collected for transfusion when required to avoid unnecessary wastage. For inter‐hospital transfer, the need for blood components during the transfer must be carefully considered by the clinical team. There is limited published experience of transfer practices for blood, but wastage of blood appears significant [17]. Clinical teams should communicate effectively with the transfusion laboratory when the decision to transfer a patient with blood is confirmed. The transfusion laboratory from the referring hospital should coordinate the transfer and traceability of blood. Blood should never be transferred without the knowledge of the transfusion laboratory. The transfusion laboratory must have a protocol in place to ensure the cold chain is maintained and evidenced during transport.

### Laboratory testing pre‐transfusion

Transfusion of blood components in patients who are haemodynamically stable continues to be mainly guided by laboratory measurements e.g. haemoglobin and platelet count. In patients with major bleeding, clinical features (e.g. heart rate, cold peripheries and prolonged capillary refill time) are often insensitive, and detection and management of coagulopathy is a critical aspect.

#### Laboratory based

Conventional coagulation tests such as prothrombin (PT), activated partial thromboplastin time (APTT) and International Normalized Ratio (INR) are well‐established, validated and familiar to most healthcare professionals. These tests were initially designed for detecting deficiencies in coagulation factors, platelet number and, for INR, directing changes in warfarin anticoagulant dosing. Disadvantages include relatively long turnaround times (average time between 27 and 77 min), which limits their utility in rapidly evolving scenarios such as major haemorrhage, their poor predictive value for bleeding (e.g. liver disease, procedure‐related) and their inability to detect the effects of direct oral anticoagulants (DOACs) or antiplatelet agents [18]. In addition, these tests are static measures that fail to capture the contributions of the endothelium, cellular and plasma component of whole blood and fibrinolysis.

#### Viscoelastic haemostatic assays (VHAs) and point‐of‐care testing

There are several VHAs systems now available on the market, with some shown to reduce peri‐operative transfusion requirements. Commonly available tests include thromboelastography (TEG™; Haemonetics, Boston, MA, USA); rotational thromboelastometry (ROTEM™; Werfen Laboratories, Barcelona, Spain); and Sonoclot (Sienco Inc., Boulder, CO, USA). A detailed review of these devices can be found elsewhere [18]. Point‐of‐care testing for haemoglobin concentration is used commonly, such as blood gas analysis or the HemoCue™ (Angelholm, Sweden), which both correlate well with laboratory measurements [19]. The activated clotting time (ACT) is also well validated and should be used routinely whenever heparin is administered, particularly in cardiac and vascular surgery [20]. These tests have been joined by newer systems such as the Quantra™ (Stago Theale, UK) utilising sonorrheometry, which measures acoustic deformation of a developing clot to measure its viscoelastic properties, and ClotPro™ (Haemonetics) to measure the effects of DOACs and antifibrinolytics [21].

Advantages of such tests include a rapid turnaround time and their ability to provide information on all phases of coagulation. Important limitations include the need for a trained user to be present, poor standardisation (apart from manufacturers' reported reference ranges), and lack of universal algorithms across different specialities [18]. Viscoelastic haemostatic assays are also recognised to be less sensitive to measuring fibrinolytic activation in trauma and should not be used to withhold the administration of tranexamic acid [18]. It is good practice to pair coagulation samples and send a second sample for conventional laboratory testing.

A systematic review assessed 12 studies in patients having cardiac surgery (n = 6835) and observed a lowering in transfusion requirements in TEG‐guided or ROTEM‐guided therapy [22]. A recent RCT in adults with major trauma (n = 396) compared standard major haemorrhage protocols using conventional coagulation tests with VHA‐guided algorithms and found no difference in the primary outcome of patients who were alive and free of massive transfusion at 24 h [23]. Current British Society of Haematology guidelines recommend the use of VHAs in patients undergoing cardiac or liver surgery, and cautiously suggest that they may also be used as part of a local algorithm to management obstetric and trauma haemorrhage, providing appropriate policies to maintain these devices are used [18].

An example of a VHA‐guided transfusion algorithm for trauma is provided in Table 1, based on a process for developing, validation and testing in a clinical trial [18]. Similarly, conventional coagulation test targets are displayed in Table 2.

**Table 1:** An example of viscoelastic thresholds used as a transfusion algorithm in trauma.

Treatment	Suggested thresholds for therapy	
	TEG	ROTEM
2 pools cryoprecipitate (equivalent to 4 g fibrinogen replacement)	FF TEG MA < 20 mm	FIBTEM CA5 < 10 mm
1 pool platelets	rTEG MA – FF TEG MA < 45 mm	EXTEM CA5 – FIBTEM CA5 < 30 mm
4 units fresh frozen plasma	rTEG MA > 65 mm plus rTEG ACT >120 s	EXTEM CA5 > 40 mm plus EXTEM CT > 80 s
Additional 1 g tranexamic acid	rTEG LY30 > 10%	EXTEM LI30 < 85%

These algorithms are suggestions and augment empiric major haemorrhage protocol therapy. This table follows data published from a clinical trial [23]. It is recommended that if similar algorithms are to be used, the algorithm is optimised for the hospital in which it is to be used. Please note do not withhold tranexamic acid therapy whilst waiting for VHA results.

ACT, activated clotting time; MA, maximal amplitude; TEG, thromboelastography; rTEG, rapid thromboelastography; FF, functional fibrinogen; ROTEM, rotational thromboelastometry.

**Table 2:** Transfusion thresholds, based on conventional coagulation tests, for major haemorrhage.

Laboratory value	Suggested target	Treatment
Haemoglobin	70–90 g.l^‐1^	Red blood cells
Platelet count	> 50 × 10^9^.l^‐1^	Platelets
	> 75 × 10^9^.l^‐1^ in obstetrics	
Fibrinogen	> 1.5 g.l^‐1^	Cryoprecipitate or fibrinogen concentrate (off label)
	> 2.0 g.l^‐1^ in obstetrics	
Prothrombin time	< 1.5 × normal	Fresh frozen plasma

### Red cell transfusion and other blood components

Details of manufacturing, storage and testing requirements for blood components discussed in this section are displayed in Table 3.

**Table 3:** Requirements for processing, storage and testing for blood and blood components in the UK.

Component	Processing	Storage and shelf‐life	Clinical practice points
Red blood cells	Manufactured by removing plasma for leukocyte‐depleted whole blood or by leucodepleting plasma reduced RBCs	Core temperature of 4 ± 2°C Maximum storage 28 days (can be extended to 35 days if adenine is added)	Time outside temperature‐controlled environment should be restricted to 30 min Transfusion should be complete within 4 h, through a 170–200 μm filter
Fresh frozen plasma	Obtained from whole blood through centrifugation or apheresis Leukodepleted Rapidly frozen to < ‐25°C to preserve coagulation factors Male donors only	Core temperature below ‐25°C for 36 months Thawed using dry ovens, microwave ovens or water bath methods Once thawed, it must never be refrozen	If delay in transfusion, FFP may be stored at 4 ± 2°C if the infusion is completed within 24 h of thawing. Pre‐thawed FFP can also be stored at 4°C for up to 5 days in patients with traumatic major haemorrhage Use 170–200 μm filter giving set
Cryoprecipitate	Manufactured by thawing FFP at 4 ± 2°C, which precipitates out FVIII; FXIII, vWF, fibronectin and fibrinogen 75% of packs should contain at least 140 mg of fibrinogen; pooled cryoprecipitate (from five donations) should contain > 700 mg	Core temperature below ‐25°C for 36 months Similar thawing processes as FFP Once thawed, it must never be refrozen	One pool = 5 single units (100–200 mls). If delay in transfusion, it can be stored at ambient temperature and used within 4 h Typical infusion rate is 10–20 ml.kg^‐1^.h^‐1^ (30–60 min for one pool) Use 170–200 μm filter giving set
Platelets	Obtained from whole blood through centrifugation or apheresis	Stored at 20–24°C under constant horizontal agitation Stored for 5 days (can be extended to 7 days with pathogen reduction)	Infusion should be started within 30 min of removal from storage Use 170–200 μm filter giving set. Avoid sets that have already been used for transfusion of RBCs

F, Factor; FFP, Fresh frozen plasma; RBC, red blood cells; vWF, von Willebrand factor.

#### Red blood cells

In patients who are haemodynamically stable and not bleeding, haemoglobin concentration is the most common clinical measurement used to guide RBC transfusion. There is now good evidence, based on an analysis of 48 randomised trials recruiting over 20,000 patients across a range of clinical settings, that a restrictive transfusion strategy (maintaining Hb between 70 and 80 g.l^‐1^) is safe and may lead to a 41% reduction in the number of patients who receive at least one RBC unit [24]. Much of this applies in clinical contexts such as orthopaedic surgery, critical care, vascular surgery, cardiac surgery, acute gastrointestinal bleeding and paediatric surgery.

Uncertainties exist in certain clinical subgroups such as those with acute coronary syndromes, acute brain injury, active cancer and older patients [25, 26, 27], and higher haemoglobin thresholds (80–100 g.l^‐1^) may be more appropriate until the results of ongoing trials are available [28]. The recent Haemoglobin transfusion threshold in traumatic brain injury optimisation (HEMOTION) trial found no evidence of a difference in unfavourable neurological outcome at 6 months in patients randomly allocated to a liberal strategy (transfusion initiated at Hb ≤ 100 g.l^‐1^) compared with a restrictive strategy (transfusion initiated at Hb ≤ 70 g.l^‐1^) [29]. However, a liberal strategy was associated with better scores on measures of motor function and quality of life. Similarly, the Transfusion Strategies in Acute Brain Injured Patients (TRAIN) found that a liberal transfusion strategy (transfusion triggered by Hb < 90 g.l^‐1^) was associated with a more favourable neurological outcome at 6 months when compared with a restrictive transfusion strategy (transfusion triggered by Hb < 70 g.l^‐1^) [30]. Two large RCTs have been published reporting on the impact of different haemoglobin thresholds on clinically relevant outcomes in patients with acute myocardial infarction (The Myocardial Ischemia and Transfusion trial (MINT) [31], Restrictive and Liberal Transfusion Strategies in Patients with AMI trial, REALITY (REALITY) [32]). Although the designs of both trials were broadly similar, the two studies reported differing directions of effect for key outcomes. MINT, the largest trial, favoured a liberal transfusion strategy with a Hb threshold of 100 g.l^‐1^. Specific guidance on these patient groups is awaited.

Single‐unit RBC transfusions are recommended in adult patients who are haemodynamically stable and without any evidence of active bleeding (or equivalent volumes calculated based on body weight for children or adults with low body weight (BMI < 18.5 kg.m^‐2^)) [33]. The haemoglobin concentration should be measured before and after every RBC unit transfused, along with a clinical assessment except when patients are actively bleeding in which case transfusion should be guided by haemodynamic response. Point‐of‐care haemoglobin measurement may be useful in limited resource settings, but laboratory measurement remains the gold standard [19].

Alternative markers for transfusion include central venous oxygen saturation; lactate clearance, acidaemia; and clinical signs (tachycardia, hypotension, ECG changes). However, these lack sensitivity and specificity to detect microcirculatory impairment, and normal values do not exclude inadequate oxygen delivery. Recent small trials have demonstrated a reduction in transfusion requirements using individualised central venous oxygen saturation, but there was no impact on clinical outcomes [34].

#### Fresh frozen plasma

Fresh frozen plasma is administered as a source of coagulation factors. The main indication for FFP is major haemorrhage, often administered in a more balanced ratio with RBCs (usually 1:1 or 1:1.5), until results of coagulation tests are available. Other indications for FFP, largely based on low quality evidence, include:

- Disseminated intravascular coagulation (DIC) with evidence of bleeding or at high risk of bleeding (e.g., planned surgery or invasive procedure)
- Reversal of warfarin anticoagulation in the presence of active bleeding if prothrombin complex is not available
- Replacement fluid for apheresis in microangiopathies (thrombotic thrombocytopenic purpura, haemolytic uremic syndrome)
- Hereditary angioedema – FFP contains C1‐esterase inhibitor

There is no good evidence to support the use of prophylactic FFP to correct abnormal coagulation tests prior to low‐risk invasive procedures in patients who are critically ill [35], although this practice still occurs. Abnormal standard coagulation tests (PT, APTT) are poor predictors of bleeding in patients who are critically ill and haemodynamically stable and do not reflect the true haemostatic status of patients with advanced liver disease [36]. The prophylactic use of FFP in elective cardiac surgery is not recommended [37]. Fresh frozen plasma should also not solely be used for volume replacement.

#### Cryoprecipitate

Cryoprecipitate is the standard concentrated source of fibrinogen in the UK and is used to treat acquired hypofibrinogenaemia.

Indications for cryoprecipitate therapy include:

- Clinically significant bleeding and a fibrinogen level < 1.5 g.l^‐1^ (< 2 g.l^‐1^ in obstetric haemorrhage).
- Fibrinogen level < 1 g.l^‐1^ and significant bleeding risk prior to a procedure, taking into consideration personal/family bleeding history, drug history, bleeding risk associated with planned procedure.
- Bleeding associated with thrombolytic therapy.
- Inherited hypofibrinogenaemia when fibrinogen concentrate is not available.

Donor and recipient blood groups should be the same for FFP and cryoprecipitate transfusion. If the patient's blood group is unknown, ABO non‐identical plasma is acceptable if it has ‘low titre’ anti‐A or anti‐B activity. Group O components should only be given to group O recipients. Any RhD group may be transfused. Fresh frozen plasma contains a very small amount of red cell stroma and sensitisation following administration of RhD‐positive plasma to an RhD‐negative individual is very unlikely to occur [38].

#### Fibrinogen concentrates

An alternative source of fibrinogen is fibrinogen concentrate. These are produced as pasteurised, lyophilised products from pooled donors that undergo purification, viral inactivation and removal processes, and do not require cross‐matching [39]. Many European countries have now moved to these as first‐line therapy for replacing fibrinogen [40]. However, clinical evidence for superiority of fibrinogen concentrates over cryoprecipitate is lacking. A recent large trial found that fibrinogen concentrate was noninferior to cryoprecipitate in patients who developed clinically significant bleeding and hypofibrinogenaemia after cardiac bypass [41]. Haemocomplettan/RiaSTAP (CSL Behring, Marburg, Germany) is the only fibrinogen concentrate globally available, and it is only licensed for use in congenital hypofibrinogenemia in the UK. More recently, Fibryga (Octapharma Ltd., Manchester, UK), a different formulation of human fibrinogen, has become available and is also licensed as component therapy for the management of uncontrolled severe haemorrhage in patients with acquired hypofibrinogenaemia during surgery.

#### Platelets

Two‐thirds of all platelets are used in patients with haematological malignancies, followed by cardiac surgery (7–10%) and critical care (5–9%) [42]. Platelet transfusions are the component most implicated in transfusion reactions, as reported by SHOT. Febrile non‐haemolytic transfusion reactions and allergic reactions are thought to occur at a frequency of 1 in 14 and 1 in 50 per‐unit transfusions, respectively and sepsis from a bacterially contaminated platelet unit is the most frequent infectious complication from any blood product [43].

Platelet transfusion may be indicated for the treatment of active bleeding (therapeutic), although severe thrombocytopenia is an uncommon complication in cases of major bleeding. In patients with severe bleeding, the following pragmatic guidance should be applied although we recognise that this is not based on high‐quality evidence:

- Maintain platelet count > 50 × 10^9^.l^‐1^
- Maintain platelet count > 100 × 10^9^.l^‐1^ in patients with multiple traumatic injuries, traumatic brain injury, or spontaneous intracerebral haemorrhage

Platelet transfusions are prescribed more commonly to treat thrombocytopaenia and thereby prevent bleeding (prophylaxis). Recent trials in neonates who were critically ill and adults with intracerebral haemorrhage associated with antiplatelet therapy have questioned the roles of platelet transfusions for either of these purposes [44, 45]. As a guide, in the absence of active bleeding, the following platelet count thresholds should be applied:

- Central venous catheter (CVC) insertion: 20 × 10^9^.l^‐1^
- Lumbar puncture: 40 × 10^9^.l^‐1^
- Insertion or removal of epidural catheter: 80 × 10^9^.l^‐1^
- Percutaneous tracheostomy: 50 × 10^9^.l^‐1^
- Major surgery: 50 × 10^9^.l^‐1^
- Neurosurgery or posterior segment ophthalmic surgery: 100 × 10^9^.l^‐1^
- Percutaneous liver biopsy: 50 × 10^9^.l^‐1^ (consider transjugular biopsy if platelet count is below this level)
- Routine prophylaxis: 10 × 10^9^.l^‐1^ (consider 10–20 × 10^9^.l^‐1^ in the presence of risk factors e.g. sepsis)

These thresholds are not based on high‐quality data and an ongoing trial is aiming to identify the optimal platelet transfusion threshold in critically ill patients requiring low‐bleeding risk invasive procedures (NIHR131822). One recent trial in the Netherlands concluded that in patients with severe thrombocytopaenia (platelet count 10–50 × 10^9^.l^‐1^) requiring CVC placement, withholding platelet transfusion was associated with a higher risk of bleeding [46]. However, this risk was largely driven by placement of subclavian CVCs in patients with haematological conditions which is not reflective of UK practice [47]. In addition, there was no evidence of increased risk in patients receiving ICU care even in those with severe thrombocytopaenia (platelet count 10–19 × 10^9^.l^‐1^). The use of platelet transfusions preprocedure when antiplatelet agents have not been discontinued is not recommended. Specific guidance for procedures related to regional anaesthesia is available elsewhere [48].

#### Prothrombin complex concentrate

Prothrombin complex concentrates (PCC) are plasma‐derived concentrates containing either three or four of the vitamin K‐dependent coagulation factors (II, VII, IX, X) [49]. Small amounts of protein C and S and antithrombin may also be present. It is produced by fractionation of pooled plasma from non‐UK donors, virally inactivated and available in lyophilised powder form which is reconstituted in sterile water immediately prior to use. Commonly available PCCs are Beriplex (CSL Behring, Marburg, Germany) and Octaplex (Octapharma Ltd., Manchester, UK). Prothrombin complex concentrates are not blood group specific. They are often stored in blood banks and not pharmacy.

The main indication for the use of PCCs is the rapid reversal of vitamin K antagonists in the context of major or life‐threatening bleeding (including intracranial bleeding). It usually reverses warfarin anticoagulation within 10–30 min. There is limited evidence to support the use of PCCs in the management of major haemorrhage not related to vitamin K antagonists. A large trial evaluating its clinical effectiveness in patients who are actively bleeding following cardiac surgery is currently ongoing (NIHR152151).

The recommended starting dose is 25 IU.kg^‐1^ [50], but this may vary according to local guidelines. An INR should be checked 30 min after dosing to ascertain the degree of correction. The indication for any further dosing should be guided by clinical and laboratory assessment of haemostatic efficacy. Due to the short half‐life of factor VII (6 h), it is essential to also give 5–10 mg of intravenous vitamin K. Important risks associated with PCC include thrombosis (arterial and venous), as demonstrated in a recent RCT [51], and hypersensitivity/allergic reactions. Where possible, patients should be consented for these.

Off‐label indications, which should be discussed on a case‐by‐case basis with a haematologist include:

- Reversal of direct oral anticoagulants (DOACs) prior to urgent/emergency surgery (i.e. rivaroxaban, apixaban);
- Reversal of anticoagulation with antithrombin agents (e.g. argatroban, dabigatran);
- Treatment of non‐life‐threatening bleeding in patients who cannot tolerate large volumes of FFP.

### Major haemorrhage

Major haemorrhage is an important cause of morbidity and mortality worldwide. Death from haemorrhage is early, with nearly 60% of deaths occurring within the first 3 h of injury. In high‐resource settings, the most common indications for massive transfusion are major surgery (61.2%) followed by trauma (15.4%) [52, 53]. The current trend is towards a pragmatic, clinically‐based definition based on the clinical status of adult patients (e.g. systolic blood pressure < 90 mmHg and/or a heart rate > 110 beats.min^‐1^) and their response to resuscitation [54]. It is important to recognise that these changes may be masked in certain patient groups (e.g. extremes of age or pregnancy). Appropriate and effective management integrates multiple factors, including: recognition; communication; avoidance of trauma‐induced coagulopathy; timely delivery of blood components; and application of definitive modalities of treatment (surgery) [55]. Surgical implementation of damage/proximal control strategies may be required for haemorrhage control to be achieved.

#### Major haemorrhage protocol

Policies should be defined in a local major haemorrhage protocol. Activation of a protocol should result in the immediate release and protocolised administration of blood components, without prior approval from a haematologist. Such protocols are encouraged to be specific to clinical areas such as the emergency department or labour ward and are designed to include robust activation and communication from bedside to laboratory. A structured form of communication (e.g. Situation, Background, Assessment, Recommendation) between clinical and laboratory areas is recommended. Their activation should also mobilise resources collaterally, such as clinical staff, portering services, blood warmers, pressure infusers and cell salvage devices [54].

A clear mechanism for the escalation of a team response and identifying individuals with sufficient seniority and experience to undertake the key roles of team leader (senior anaesthetist) and co‐ordinator, are essential to the process enabling a single point of contact with the laboratory and other support services. Deactivation of major haemorrhage protocols should not be forgotten, as delays in doing so may lead to blood wastage and prevent resumption of other laboratory and clinical services. Major haemorrhage protocols should be reviewed at least annually, or whenever there are changes in guidance or new evidence becomes available to suggest change in practice.

#### Initial resuscitation

Most major (non‐obstetric) haemorrhage packs will contain four units of RBCs and four units of FFP (equivalent to 15–20 ml.kg^‐1^ in a standard adult); platelet concentrate may also be provided [54]. Administration should be via wide‐bore intravenous access, or via intra‐osseous access until the former can be obtained.

Group O RBCs should be available immediately and transfused if haemorrhage is life‐threatening. Group O RhD negative and K‐negative RBCs should be prioritised in women of childbearing potential (< 50 years), children and when the patient's sex is unknown. Group O RhD positive RBCs should be issued for adults who do not have childbearing potential [54]. In certain sites (e.g. elective surgery/high volume, low capacity hubs) only Group O RhD positive RBCs are stored. While the likelihood of transfusion is low, patient selection for such sites is important (i.e. patients without RBC antibodies). Group‐specific RBCs should be rapidly made available (within 15 to 20 min) of the laboratory receiving a correctly labelled crossmatch sample and being informed of the emergency requirement for blood.

#### Haemostatic resuscitation

The aims of haemostatic resuscitation are to: restore and sustain normal tissue perfusion; maintain and regularly monitor haemostasis; and avoid the lethal triad of hypothermia, acidosis and coagulopathy [54, 55]. Electrolyte abnormalities such as hyperkalaemia and hypocalcaemia (aim for ionised calcium > 1.0 mmol.l^‐1^) should be treated promptly.

Current evidence from RCTs does not demonstrate clear superiority of VHAs over conventional coagulation tests on clinically important outcomes but may reduce peri‐operative blood component transfusion requirements [56]. Both VHAs and conventional coagulation tests have their advantages and limitations (see Section on ‘Laboratory testing pre‐transfusion’). What is perhaps more important is the process of repeated testing, and comparisons and reassessments between serial tests, rather than the results of isolated/standalone tests to guide decision making.

### Prehospital trauma

Prehospital management of major traumatic haemorrhage is evolving constantly. Anaesthetists are involved increasingly in the care of these seriously ill and injured patients that need airway protection, ventilatory support or transfusion support before they arrive in hospital [57]. Although patterns of blood component use are variable, time to initial transfusion is perhaps more important in trauma [58].

Approximately a quarter of patients with severe trauma will present with coagulopathy [59], defined typically by abnormalities of prothrombin time; this is fatal in 30–50% of cases. Early transfusion has been advocated to treat and/or mitigate trauma‐induced coagulopathy. Observational data from the US military have shown that prehospital transfusion within minutes of injury was associated with greater 24‐h and 30‐day survival than delayed transfusion or no transfusion [60]. Similarly, UK civilian data have shown that prehospital combined plasma and RBC transfusion was associated with lower odds of death at 24 h when compared with RBC transfusion alone [61]. However, such studies have important recognised limitations such as survivorship bias and residual confounding. Closer inspection of trial data suggests that differences in mortality rates may be related to differences in transport times, modes of transport, patient characteristics (e.g. age), illness severity and components administered. A large UK trial, aiming to enrol 848 participants, is currently evaluating the use of pre‐hospital (via air ambulance) whole blood administration in patients requiring pre‐hospital blood transfusion to treat major traumatic haemorrhage (ISRTCN23657907).

Low fibrinogen levels (< 1.5 g.l^‐1^) are also common in patients who have suffered trauma and are associated with poor clinical outcomes [62]. The CRYOSTAT‐2 trial randomly allocated 1604 patients to standard care, which was the local major haemorrhage protocol, or early and empirical high‐dose cryoprecipitate (three pools of cryoprecipitate, equivalence to 6 g of fibrinogen) within 90 min of randomisation and 3 h of injury [63]. The authors found no evidence of an effect on the primary outcome of all‐cause mortality at 28 days. In a prespecified subgroup analysis, 28‐day mortality was higher in patients with penetrating trauma who were allocated to the cryoprecipitate (16.2% vs. 10.0%, odds ratio (95%CI) 1.74 (1.20–2.51), p = 0.006) but the underlying mechanisms of this finding are unclear. The use of tranexamic acid, administered within 3 h of injury, reduces mortality in patients who are bleeding after trauma [64] and in those with mild‐to‐moderate brain traumatic brain injury [65]. Gruen et al. recently reported the results of a large RCT, in advanced trauma systems, evaluating the safety and efficacy of prehospital tranexamic acid in patients with severe trauma who were at risk of trauma‐induced coagulopathy [66]. The authors observed a lower 28‐day mortality rate in patients who received tranexamic acid, but no difference in the primary outcome of survival with a favourable functional outcome at 6 months [66].

Key principles include:

*Early haemorrhage control*

Ensure the clinical course is aimed towards haemorrhage control. Use temporary haemostatic devices (pressure, tourniquets, etc.) followed by surgery or interventional radiology for control of haemorrhage [53, 54].

*Blood pressure management*

Do not attempt to normalise blood pressure during active haemorrhage. Achieve a lower acceptable blood pressure with volume resuscitation alone. This may need to be modified in patients with identified, or suspected traumatic brain injury, where brain injury is the dominant condition. During uncontrolled haemorrhage, implement major haemorrhage protocols for volume resuscitation [53]. Crystalloids should only be given where there is profound hypotension and no imminent availability of blood components. The use of vasopressors should be avoided during active haemorrhage [53, 54].

*Target trauma‐induced coagulopathy*

Deliver blood components empirically at first and, once in hospital, use laboratory coagulation tests or VHAs to guide therapy as soon as available. Give tranexamic acid (Table 4) immediately, but avoid if more than 3 h from injury, unless there is ongoing evidence of hyperfibrinolysis from point of care testing [54]. Whilst haemorrhage is being controlled, protocolised administration of RBC and FFP in a ratio of 1:1 should be used to replace circulating volume [54]. Consider the administration of cryoprecipitate (two pools) and platelets (one adult therapeutic dose) until test results are available and bleeding is controlled. Administer platelets and cryoprecipitate as per major haemorrhage protocols.

**Table 4:** Suggested doses of tranexamic acid according to clinical setting.

Indication	Dosing schedule
Postpartum haemorrhage	1 g i.v. (over 10 min) within 3 h of bleeding onset
	If bleeding continues after 30 min, or it stopped and restarted within 24 h of the first dose, given a second dose of 1 g i.v.
Major trauma, including mild to moderate TBI	1 g i.v. (over 10 min) within 3 h of injury, followed by a maintenance infusion of 1 g over 8 h OR 2 g i.v. (over 20 min) as single bolus within 3 h of injury
Major surgery	
‐ Non‐cardiac	1 g i.v. (over 10 min) prior to skin incision and end of surgery
‐ Cardiac	50–100 mg.kg^‐1^ i.v. over 30 min after induction of anaesthesia
‐ Orthopaedics	1 g i.v. (over 10 min) prior to skin incision
Gastrointestinal bleeding	Not recommended
Paediatrics	
‐ Major trauma/non‐cardiac surgery	15 mg.kg^‐1^ i.v. (max 1 g) over 10 min followed by 2 mg.kg^‐1^.h^‐1^ infusion until closure or reduction of risk of haemorrhage

i.v., intravenous; TBI, traumatic brain injury.

Once haemorrhage control is achieved, blood product administration should be guided by regular laboratory and/or VHA testing. Target endpoints for resuscitation include: pH > 7.2; and normalising base deficit and lactate.

### Critical care

Anaemia is common in patients who are critically ill and is associated with poor short and long‐term clinical outcomes [67, 68, 69]. Haemoglobin drops by a mean (SD) of 0.52 (0.69) g.l^‐1^ per day whilst on ICU [70]. Approximately 30–40% of patients who are critically ill will have moderately severe anaemia (Hb < 90 g.l^‐1^) at some point during their ICU stay [71]. Patients who are critically ill display the hallmarks of anaemia of inflammation characterised by disturbed iron homeostasis, impaired erythropoiesis and reduced red cell survival [72]. This is often exacerbated by haemodilution and blood loss from phlebotomy, extracorporeal circuits and/or surgery [71].

The current mainstay of treatment for anaemia in patients who are critically ill is RBC transfusion. A restrictive transfusion threshold of Hb < 70 g.l^‐1^ is recommended in most patients, including those with ARDS and septic shock [73, 74]. Uncertainties on the optimal transfusion remain in patients who are critically ill with acute coronary syndromes or ischaemic heart disease (see Section on ‘Red blood cells’). Patients with solid organ and haematological malignancy and who are critically ill often require blood components. However, the evidence in these groups is largely from single‐centre RCTs [26, 75, 76] and there is no agreed consensus on the optimal transfusion threshold.

Available evidence does not support the routine use of iron therapy and/or erythropoietin to treat anaemia in patients who are critically ill [77]. Although improvements in haemoglobin have been demonstrated, this has not translated into reduced blood usage or improvement in clinical outcomes. Large trials, targeted at treating anaemia in patients recovering from critical illness, are underway. Strategies to minimise iatrogenic anaemia should be encouraged. These include limiting blood sampling by using small‐volume tubes and avoiding standing orders, using blood conservation devices and appropriate management of medications likely to cause anaemia and/or bleeding. A large cluster, multicentre RCT found that the use of small‐volume tubes in the ICU may decrease RBC transfusions without affecting laboratory analysis [78], and their use is now recommended by international guidelines [79].

### Obstetrics

Estimating blood loss at delivery is inaccurate [80]. Every effort should be made to measure blood loss volumes cumulatively, with volumetric and gravimetric techniques after all deliveries so escalation is based on actual rather than estimated blood loss. Early recognition of bleeding incorporated into a clear escalation plan of obstetric intervention and involvement of the multi‐professional team reduces the use of blood components [81]. In an international RCT, a multicomponent intervention consisting of early detection of postpartum haemorrhage (PPH), with a calibrated blood‐collection drape and a bundle of first‐response treatments (uterine massage, oxytocic drugs, tranexamic acid, intravenous fluids, examination and escalation) led to a lower risk of severe PPH, death from bleeding and laparotomy for bleeding when compared with usual care [82], whilst also demonstrating cost‐effectiveness [83].

As soon as abnormal bleeding is recognised (> 500 ml after a vaginal delivery and > 1000 ml after a caesarean delivery) the obstetrician, anaesthetist and senior midwife should attend to the mother. Blood should be taken for full blood count, clotting studies, group and screen, and a venous blood gas for rapid lactate and haemoglobin measurement. A lactate > 2 mmol.l^‐1^ is an indicator of shock but a normal haemoglobin can be falsely reassuring before resuscitation. A clotting screen, lactate and haemoglobin should be repeated for every 500 ml of continuing blood loss or for clinical concerns to ensure a timely response to an abnormal haemoglobin result or coagulopathy. If available, a paired sample should be taken for VHA. Cell salvage is recommended if abnormal bleeding occurs during caesarean section, and a leucocyte filter should be used for autotransfusion of processed blood [84].

Hypofibrinogenaemia (defined as Clauss fibrinogen < 2 g.l^‐1^) is the most common factor deficiency in PPH, occurring in 5% of PPHs at 1000 ml and 17% at 2500 ml [85]. A laboratory Clauss fibrinogen of < 3 and especially < 2 g.l^‐1^, with ongoing bleeding, is associated with progression to massive obstetric bleeding (> 2500 ml) [86, 87]. There is some evidence that early identification and treatment of hypofibrinogenaemia can reduce the progression from major to massive PPH. Prolongation of the PT and APTT is less common, affecting 1% of haemorrhages at 1000 ml and typically becomes prolonged at a bleed volume > 4000 ml. Any prolongation of the PT and APTT in a pregnant person above the normal nonpregnant range may indicate factor depletion and should be managed with FFP [88]. Severe early hypofibrinogenaemia (fibrinogen < 2 g.l^‐1^) occurs in around 5% of bleeds at 1000 ml and is associated with abruption, amniotic fluid embolus and severe bleeding with sepsis and pre‐eclampsia [83]. It can also occur unexpectedly in association with any obstetric aetiologies. Early use of cryoprecipitate or fibrinogen concentrate before RBC may be required, with repeated administration if bleeding is ongoing.

Postpartum haemorrhage associated with atony or trauma is less likely to be associated with haemostatic impairment unless the diagnosis is delayed, and protocol‐led use of blood components will lead to excessive transfusion of FFP in most cases [89]. If coagulation tests are not known, then FFP should be withheld until four units of RBC have been given, unless the traumatic or atonic bleed is associated with other causes of early coagulopathy diagnosed on an early coagulation or VHA screen. If no coagulation results are available and bleeding is ongoing, then, after four units of RBC, four units of FFP should be infused and 1:1 ratio of RBC:FFP transfusion maintained until the results of haemostatic tests are known. A recent feasibility trial provided encouraging clinical data to support the use of cryoprecipitate before FFP in first‐issued haemorrhage packs, but confirmatory data are needed [90].

Platelet transfusion is required rarely unless PPH is > 5000 ml or platelet count is < 100 × 10^9^.l^‐1^ from another cause prior to the PPH [91]. Platelets should be transfused when the platelet count is < 75 × 10^9^.l^‐1^.

Monitoring haemostatic function in obstetric haemorrhage is important because coagulopathy is unpredictable, and the aetiology is not always clear at the start of the PPH. The potential advantages and limitations of VHAs have been discussed previously. These can be used in all hospital‐based maternity settings [87] and their use across the UK is increasing with a reported 30 maternity units already using them routinely, and a further 36 obstetric units set to use them as part of a stepped‐wedge cluster randomised trials (NIHR152057). Interpretation should focus on the fibrinogen assay and replacement with fibrinogen concentrate or cryoprecipitate prioritised. Laboratory and VHA results should be interpreted within the clinical situation, repeated if bleeding is ongoing, and blood components withheld if bleeding has stopped [92]. Hyperfibrinolysis is common in early PPH [89]. Tranexamic acid reduces total blood loss and should be given if PPH is identified (> 500 ml after a vaginal delivery and > 1000 ml after a caesarean delivery) [93] (Table 4).

### Paediatrics

Evidence to guide the use of blood components in children undergoing surgery is limited, and often extrapolated from adult practice. Serious Hazards of Transfusion has identified a higher incidence of serious adverse events in children receiving blood components [1]. Transfusion in children is largely the same as transfusion in adults, with adaptations of volumes and dose. Normal range for haemoglobin concentrations in children are shown in Table 5. Restrictive thresholds (< 70 g.l^‐1^) for RBC transfusion are appropriate for almost all children over 3 months of age. Higher transfusion thresholds are often applied to neonates and children with congenital heart disease. Although thresholds are not clearly defined, there is evidence that transfusion volumes can be reduced in these patients by applying moderately restrictive thresholds without adverse effect on outcomes [94]. In children who are critically ill, haemodynamically stable and not bleeding, maintaining a haemoglobin >70 g.l^‐1^ is recommended, including situations where oxygen delivery is limited (e.g. septic shock, acute traumatic brain injury and post‐cardiac surgery) [95]. Neonates should receive components specified for neonatal use, including cytomegalovirus‐negative blood components [96].

**Table 5:** Normal ranges for haemoglobin in children [97].

Age	Haemoglobin (g.l^‐1^)
Birth	140–240
2 weeks	134–198
4 weeks	134–198
2–6 months	94–130
6–12 months	111–141
1–6 years	115–140
6–12 years	115–155
12–18 years (girl)	120–160
12–18 years (boy)	130–170

The volume of blood needed should be modified depending on the size of the patient. It is recommended that blood in children should be prescribed in volume rather than number of units. A single pre‐operative RBC transfusion of 10 ml.kg^‐1^ should increase the Hb by approximately 20 g.L^‐1^ [96]. A formula used commonly to calculate the volume of RBCs to transfuse is provided below [98]:

$$\text{Volume}\left(\mathrm{ml}\right)=\frac{\text{Desired}\;\mathrm{haemoglobin}\left(g.l^{-1}\right)-\text{actual}\;\mathrm{haemoglobin}\left(g.l^{-1}\right)\times \text{weight}\left(\mathrm{kg}\right)\times 4}{10}$$

Other blood components should be given as follows [96]:

- Cryoprecipitate: 5–10 ml.kg^‐1^
- Platelets: 10–20 ml.kg^‐1^
- FFP: 10–15 ml.kg^‐1^
- Fibrinogen concentrate: 70 mg.kg^−1^ (max 2 g); this can be increased to 100 mg.kg^−1^ in severe bleeding (with hypofibrinogenaemia) where doses > 2 g have been used

Tranexamic acid should be used in children undergoing major surgery likely to be associated with major blood loss such as cardiac surgery, major scoliosis surgery and craniosynostosis surgery, often as part of a multi‐component patient blood management intervention [99, 100] (Table 4). It can also be used in major traumatic haemorrhage as part of a massive haemorrhage protocol. Tranexamic acid dosing is uncertain and other dosing schedules (including higher infusion rates) are also acceptable and in current use. Children > 12 years old should receive the adult dose.

### Cardiac surgery

Anaemia is associated with poor outcomes in patients undergoing cardiac surgery [101]. Elective cardiac surgery should not be undertaken in patients with anaemia until the cause has been investigated and treated appropriately. Detailed guidance is now available on the investigation and management of pre‐operative anaemia [9].

Pooled data from eight RCTs showed no evidence of an impact on clinically important outcomes when a restrictive RBC transfusion policy was followed when compared with a liberal one [25]. A transfusion threshold of 75 g.l^−1^ would be reasonable as this is threshold that was used in most of the included trials. Markers of adequate oxygen delivery such as lactate, cerebral near infrared spectroscopy values and central venous oxygen saturation can be considered to guide more individualised transfusion decisions [34]. Patient blood management guidelines should be implemented throughout the patient pathway. The routine use of cell salvage to reduce intra‐operative blood loss and RBC transfusion in cardiac surgery is recommended [10].

A meta‐analysis of 12 studies reported that continuing antiplatelet therapy (aspirin, clopidogrel) until the time of cardiac surgery was associated with increased blood loss but carried a low risk of surgical re‐exploration for bleeding [102]. The authors concluded that in patients at a high risk of stent thrombosis, this may be acceptable. Stopping antiplatelet therapy should therefore be undertaken in the context of the risk and benefit ratio to each individual patient.

#### Considerations for cardiopulmonary bypass

Interaction of blood with foreign surfaces leads to a derangement in coagulation processes, dilutional hypofibrinogenaemia and platelet function. Perfusionists endeavour to minimise RBC transfusion intra‐operatively due to its association with increased morbidity and mortality [103, 104]. Currently, there is no fixed level haemoglobin level to guide the decision for transfusion; however, a haemoglobin < 75 g.l^‐1^ is clinically accepted [105]. In terms of perfusion, refinement of inline monitoring systems such as the CDI 500 (Terumo UK Ltd., Camberley, UK) integrated into circuits have allowed for close attention to factors such as venous saturation and oxygen delivery, improving blood management and conservation. Miniaturising cardiopulmonary bypass circuits attenuates the inflammatory response to bypass and improves haemodilution, reducing transfusion requirements [106]. The process of conventional ultrafiltration during bypass can have a positive impact on removing excess volume and raising haematocrit in both adult and paediatric cardiac surgery [107]. Moreover, modified ultrafiltration, more frequently performed following termination of paediatric bypass, can raise haematocrit as well as improve pulmonary compliance [108].

Challenges arise when patients are unwilling to receive allogeneic blood due to religious or other beliefs. An individual discussion should take place to determine what is acceptable to the patient. Cell salvage is used frequently to process the patient's own blood during surgery. Retrograde autologous priming to remove a proportion of the clear priming volume has been shown to reduce the need for transfusion on bypass over other methods such as acute normovolemic haemodilution [109, 110].

### Gastrointestinal bleeding

Acute upper gastrointestinal bleeding is common with an estimated incidence of 134 per 100,000 population, equating to approximately one presentation every 6 min [111]. The commonest causes include peptic ulcer disease, varices and malignancy [112]. Despite advances in medical care, mortality over the past two decades has remained high at 10%. Initial management includes fluid resuscitation if haemodynamically unstable, transfusion support, risk stratification, pharmacological therapies (e.g. terlipressin for those with suspected cirrhosis/variceal bleeding) and timely access to endoscopy or interventional radiology [111]. Anaesthetists are often involved in the care of these patients for airway protection in those with ongoing haematemesis and altered mental and respiratory status.

High certainty evidence recommends a transfusion threshold of 70 g.l^‐1^, aiming for a target of 70–100 g.l^‐1^ [113]. A higher threshold should be considered in patients with cardiovascular disease. The role of tranexamic acid in gastrointestinal bleeding has recently been questioned with the results of a large, pragmatic trial showing no reduction in mortality and increased risk of venous thrombosis [112]. Its use in this setting is therefore not recommended. There are limited data characterising changes in coagulopathy in patients with gastrointestinal bleeding, and the limited role of conventional coagulation tests (e.g. PT, INR) in predicting bleeding, particularly in patients with liver disease, is increasingly recognised. In patients who are actively bleeding/haemodynamically unstable, adherence to standard major haemorrhage protocols and referral to critical care are recommended [111].

The incidence of lower gastrointestinal bleeding is increasing and accounts for 3% of emergency surgical referrals with an inpatient mortality of 3.4%, which rises to 18% in patients who develop lower gastrointestinal bleeding while already hospitalised [114]. The most common cause of lower gastrointestinal bleeding in the UK is diverticular disease, followed by benign anorectal conditions (e.g. fissures, haemorrhoids and rectal ulcers). Management includes risk stratification, interventions to diagnose and treat bleeding (e.g. colonoscopy, interventional radiology, endoscopic therapy and surgery), reversing anticoagulant therapy in life‐threatening haemorrhage and transfusion support. Nearly one‐third of patients with lower gastrointestinal bleeding will receive a RBC transfusion but up to 80% of these may be inappropriate or unnecessary [114]. Evidence for RBC transfusion thresholds is limited and there are no RCT data. Therefore, similar thresholds to those recommended in patients with upper gastrointestinal bleeding are recommended [114].

### Drugs that affect coagulation

#### Anticoagulants

Large numbers of patients take either anticoagulants or antiplatelet agents for the prevention of thrombosis. Increasing numbers now take a direct thrombin or Xa inhibitor rather than warfarin. The management of these drugs in the peri‐operative setting is a common problem, necessitating the balance of bleeding risk with the risk of thrombosis. There are two scoring systems that can be used to quantify the risk of thrombosis (CHA_2_DS_2_‐VASc) and bleeding (HAS‐BLED) in patients who are anticoagulated for nonvalvular atrial fibrillation [115].

#### Direct oral anticoagulants

Direct oral anticoagulants (DOACs) have more predictable pharmacodynamics, a faster onset of action and a shorter half‐life than warfarin and require less frequent monitoring of plasma levels [116]. There are currently four drugs available – three anti‐Xa drugs (apixaban, edoxaban and rivoroxaban) and one direct thrombin inhibitor (dabigatran). These drugs are used: in the management of patients with atrial fibrillation; after stroke and transient ischaemic attacks; and prophylaxis/management of venous thromboembolism. Direct oral anticoagulants are now more commonly used than warfarin in these settings [117]. They are not licensed for use in patients with prosthetic (metal) heart valves based on RCT data demonstrating increased rates of thromboembolic complications when compared with warfarin therapy [118, 119].

**Figure 1:**
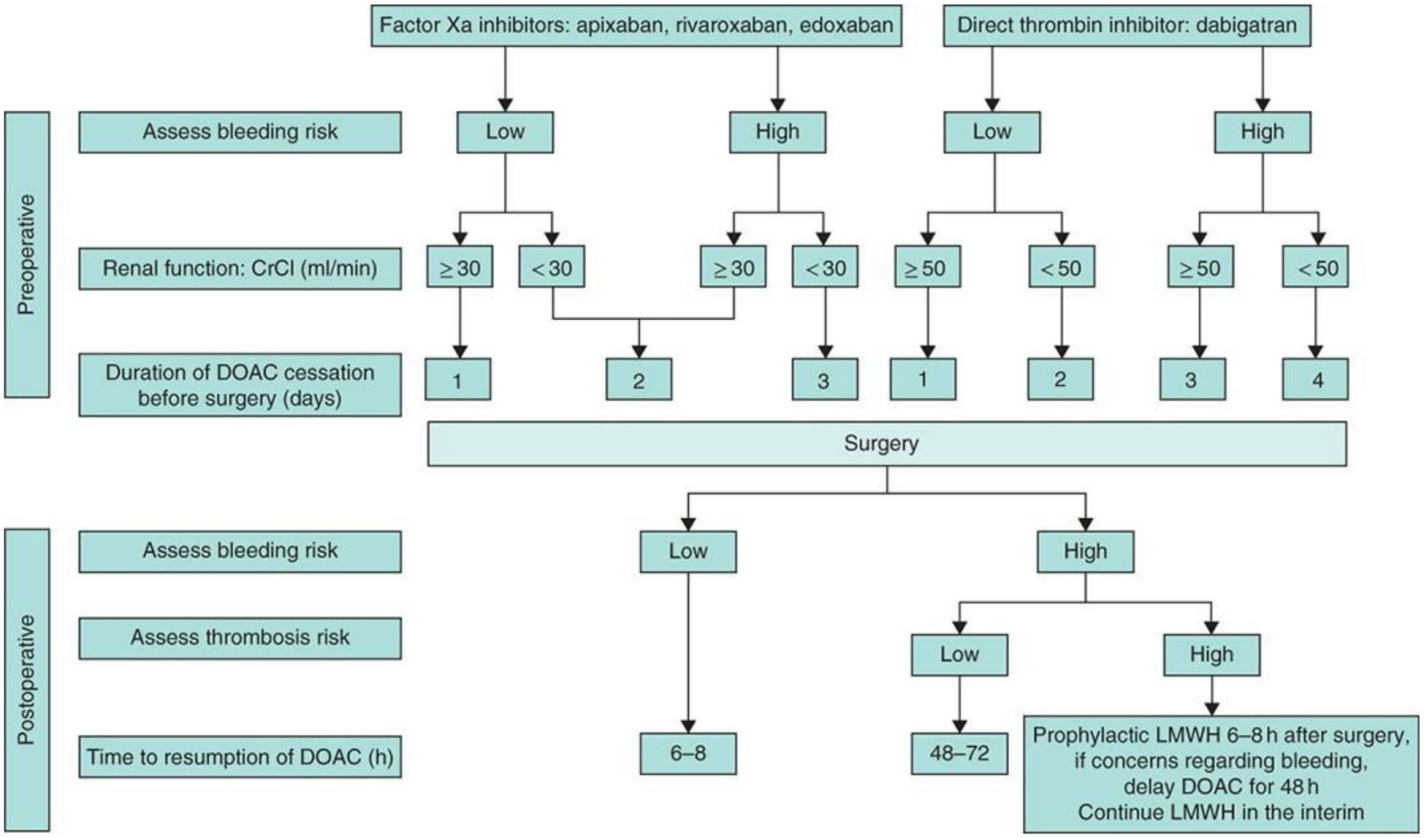
Suggested algorithm for peri‐operative management of directly acting anticoagulants (Reproduced with permission). CrCl, creatinine clearance; DOAC, direct oral anticoagulant; LWMH, low molecular weight heparin.

The approach to the peri‐operative management of patients taking DOACs is based on an approximate calculation of the half‐life of the drug, considering renal function. This is combined with the bleeding risk of the proposed procedure and the patient's individual risk factors for thrombosis and bleeding (Fig. 1). If an anticoagulant effect cannot be excluded, neuraxial anaesthesia should be avoided. Bridging anticoagulation (usually with low molecular weight heparin) is not required except in patients deemed to be at high risk on peri‐operative thromboembolism (e.g. with recent (< 3 months and especially 1 month) history of pulmonary embolism or deep venous thrombosis; severe thrombophilia (deficiency or protein C, protein S or antithrombin); antiphospholipid antibodies; or active cancer associated with a high thromboembolism risk) [120].

#### Emergency surgery

Prothrombin complex concentrates should not be used routinely in patients taking DOACs prior to emergency surgery. Andexanet alfa and idarucizumab are targeted reversal agents [121]. Andexanet alfa is a modified recombinant inactive form of human factor Xa, which binds and sequesters to factor Xa inhibitors [121]. It should be used to reverse apixaban, rivaroxaban or edoxaban prior to emergency invasive procedures and surgery where the bleeding risk is considered significant. It also binds to tissue factor pathway inhibitor, reducing its activity and subsequently increasing thrombin formation. Cohort studies have reported the incidence of thrombotic events within the first 30 days of treatment to be around 10% [122]. However, when any form of anticoagulation was commenced within 30 days post‐andexanet therapy, no thrombotic events occurred. This finding supports prompt resumption of anticoagulation after major bleeding in these patients once safe to do so.

Current indications for andexanet alfa are: treatment of severe gastrointestinal haemorrhage; and limitation of haematoma size in intracranial haemorrhage. However, as andexanet alfa can reverse the anticoagulant effects of unfractionated heparin, it should not be used in patients undergoing surgery where some degree of anticoagulation is required (e.g. requirement for cardiopulmonary bypass and non‐cardiac vascular surgery).

Idarucizumab, a specific antagonist to dabigatran, can be used to reverse dabigatran effect rapidly. There are newer drugs in development such as ciraparantag, which binds noncovalently to heparin, LWMH and DOACs, that are undergoing early phase trials [123].

#### Warfarin

Warfarin has a half‐life of approximately 36 h and so should therefore be stopped 5 days before elective surgery to ensure haemostasis has returned to normal. The INR should be measured the day prior to surgery. If the risk of thrombosis is high, bridging can be used to reduce the thrombotic risk, although it is worth noting there is little proven benefit and a possible risk of increased peri‐operative bleeding in patients taking warfarin for atrial fibrillation [124].

Individualised decisions are recommended whether bridging is necessary in certain situations; in particular, those patients taking warfarin for mechanical heart valves require special consideration. The type of valve (bileaflet vs. caged ball and tilting disc), valve position (aortic vs. mitral) and patient risk factors (such as previous stroke or transient ischaemic attack, atrial fibrillation) should be considered; however, in general all patients with mechanical heart valves (with the exception of bileaflet aortic valve with no other risk factors) should have bridging anticoagulation [125].

For patients with acute VTE the risk of recurrence without anticoagulation is very high in the first 3 months, and surgery will increase the risk further; therefore, bridging should be considered (Table 6). When a patient is more than 3 months from an acute event the risk of recurrence is much lower and postoperative prophylactic dose LMWH should be sufficient. For patients with AF, the CHA_2_DS_2_‐VASC score may be used to predict stroke risk and help select those who would benefit from bridging [124]. Patients with AF who have a score of ≤ 4 and who have not had a stroke or TIA in the last 3 months should not receive bridging. Patients who have had a stroke or TIA within the last 3 months, or who have a previous stroke/TIA with three or more risk factors (hypertension, age > 75 years, diabetes or congestive cardiac failure), should be considered for bridging [125].

**Table 6:** Peri‐operative bridging warfarin anticoagulation for major surgery with high thrombotic risk.

Day 6 pre‐op	Days 2–5 pre‐op	Day 1 pre‐op	Day of surgery	Post‐op
Last warfarin dose	Treatment dose LMWH	Full or half treatment dose LMWH (Full dose if > 24 h before surgery)	No treatment Consider mechanical thromboprophylaxis	Prophylactic LMWH as minimum. In high‐risk patients give treatment dose until warfarin is therapeutic
		Check INR		

INR, International Normalised Ratio; LMWH, Low molecular weight heparin.

#### Emergency surgery

If surgery can wait for 6–8 h, then 5 mg of intravenous phytomenadione (vitamin K) can restore coagulation factors although this may preclude warfarinisation for a few days subsequently; if this is not possible, anticoagulation can be reversed with 25–50 iu.kg^‐1^ of PCC [126].

#### Antiplatelet drugs

Antiplatelet drugs are used in the secondary prevention of cardiovascular disease. They may be taken individually or as dual antiplatelet therapy (DAPT), usually following acute coronary syndromes or after coronary artery stenting. Aspirin inhibits the production of thromboxane. If taken alone it should be continued for most procedures until the day before surgery as most evidence suggests it does not increase bleeding [127], except for surgeries with a particularly high bleeding risk or surgery in a confined space (brain, posterior chamber of the eye, medullary canal) where it should be discontinued 5 days before the procedure. Adenosine diphosphate (ADP) receptor (P2Y12) antagonists (including clopidogrel, prasugrel and ticagrelor) are thienopyridines. They should be discontinued 5–7 days pre‐operatively [128]. Consideration should be given to the use of haemadsorption filters if urgent or emergency cardiopulmonary bypass is undertaken in patients with P2Y12 inhibitors [129].

#### Antiplatelet drugs in patients with coronary stents having non‐cardiac surgery

The management of these drugs in patients with coronary stents in situ is more challenging. Dual antiplatelet therapy is recommended for at least 4 weeks after the insertion of a bare metal stent and for 12 months after a drug‐eluting stent. The risk of peri‐operative adverse cardiac events reduces as time passes following the insertion of the stent. Very low bleeding‐risk procedures can be undertaken without stopping DAPT. Low bleeding‐risk procedures in patients with low thrombotic risk may be undertaken with discontinuation of the ADP‐receptor antagonist (5–7 days pre‐operatively) with aspirin continuing.

Ideally, elective surgery in patients deemed to be at high thrombotic risk (within 6 months of stent insertion) should be deferred until they are lower risk. If this is not possible it should proceed on aspirin with temporary discontinuation of the ADP‐receptor antagonist [125]. Bridging with a parenteral short‐acting glycoprotein IIb/IIIa inhibitor, such as tirofiban or eptifibatide, during the period of ADP receptor antagonist withdrawal [130] can also be considered in conjunction with cardiology input.

For emergency surgery, management depends on the antiplatelet agent and when the last dose was taken. Platelet transfusions have been proven to improve haemostasis [131]. Platelet function testing may be helpful to determine the degree of platelet inhibition. Neuraxial anaesthesia should be avoided when ADP antagonists have been taken within 7 days, although aspirin is generally thought to be safe to continue.

### Drugs that decrease blood loss

#### Tranexamic acid

Tranexamic acid is a synthetic derivative of the amino acid lysine that inhibits plasminogen activation, thus inhibiting fibrinolysis. It has been proven to reduce bleeding in most surgical settings including cardiac and major non‐cardiac surgery, major trauma and postpartum haemorrhage [64, 65, 66, 93, 132, 133, 134]. A meta‐analysis of 216 trials (125,550 participants) across a range of clinical settings found no evidence of an increased risk of thromboembolic complications associated with the use of tranexamic acid, supporting the general safety of the drug [135]. More recently, another meta‐analysis focusing on patients undergoing non‐cardiac surgery (191 RCTs, 40,621 participants) also found no evidence of an increased risk of cardiovascular thromboembolic complications, seizures or mortality at 30 days with tranexamic acid use [136]. A suggested dosing schedule for different clinical indications is provided in Table 4. It should be used with caution in patients with massive haematuria (if risk of ureteric obstruction) and in patients on oral contraceptive pills (risk of thrombosis) and is contraindicated in disseminated intravascular coagulation.

#### Aprotinin

Aprotinin is a serine protease inhibitor which has been widely used in cardiac surgery as an antifibrinolytic agent to minimise patient bleeding. It was withdrawn from the European market in 2008 for safety reasons but was reintroduced in 2012 with narrow licencing indications, specifically isolated coronary artery bypass graft surgery in high‐risk patients. However, in clinical practice, its predominant use appears to be outside its licence, mainly being used in patients likely to bleed during major cardiac surgery such as acute aortic dissection and surgery in patients with infective endocarditis [8]. This is supported by observational data where aprotinin was associated with a lower incidence of massive bleeding and reduced mortality in high‐risk cardiac surgery, but not in low to moderate‐risk patients [137].

#### Desmopressin

Desmopressin (or DDAVP or 1‐deamino‐8‐D‐arginine vasopressin) is a pro‐haemostatic drug that is used commonly for patients with inherited bleeding disorders [138]. Desmopressin stimulates release of von Willebrand factor (vWF) from the endothelium which then binds platelets to collagen through GP1b receptors and to other platelets through GPIIb/IIIa receptors [138]. It has been considered as an alternative to platelet transfusions [139]. Desmopressin in a dose of 0.3 μg.kg^‐1^ may be considered in patients presenting for surgery with acquired or inherited vWF deficiency.

The use of desmopressin has been evaluated in the peri‐operative setting where it may lead to a small reduction in blood loss and volume of RBCs transfused in patients undergoing cardiac surgery [140]. However, these differences are unlikely to be of clinical importance. Patients that may benefit from desmopressin include those with platelet dysfunction secondary to cardiopulmonary bypass or recent antiplatelet therapy prior to cardiac surgery, but the certainty of the evidence is low [141, 142]. A small feasibility RCT found no evidence of an efficacy signal for desmopressin on raising vWF levels or other markers of haemostasis in patients who are critically ill [143].

In patients on antiplatelet drugs who experience intracranial haemorrhage, a multicentre trial demonstrated feasibility and safety of desmopressin administration, but confirmatory data are needed [144].

## Discussion

These guidelines have been made using the best available contemporary evidence and pragmatic expert consensus involving multiple stakeholders. The expanding number of RCTs evaluating different transfusion thresholds for RBCs and platelets will continue to inform best practice in adults and children across a range of clinical conditions. Good transfusion practice should not solely rely on haemoglobin thresholds but also incorporate the clinical context, patient comorbidities, clinical signs and symptoms, rate of bleeding and patient preferences.

Major bleeding is an important cause of death in surgical patients [145]. The 7th National Audit Project found that major haemorrhage was the leading cause of peri‐operative cardiac arrest [145]. Healthcare professionals involved in the care of patients at risk of major peri‐operative blood loss should also be aware of the current prophylactic and therapeutic options available to them. High‐quality evidence supports the safety and efficacy of tranexamic acid in preventing surgical bleeding but according to the 2023 NHS national comparative audit, around one‐third of patients who were potentially eligible to receive tranexamic acid did not receive it [146]. Uptake of tranexamic use is particularly low in certain surgical specialities (e.g. vascular surgery). Recommendations from the Infected Blood Inquiry report include adding tranexamic acid on surgical checklists, a requirement for medical directors to report to their boards and the chief executive of their Trust on the extent of it us, and the board to report to NHS England annually as to the percentage of eligible operations which have involved its use (which should be above 80% [147]).

Our guideline has limitations. We synthesised data qualitatively and did not perform any statistical analysis. Some recommendations were adopted from guidance produced by others. Our recommendations may also not applicable to other healthcare systems, however our aim was to generate a UK‐centric guideline.

In summary, we developed a multidisciplinary guideline aiming to improve transfusion safety and prevent major bleeding. Given the dynamic nature of the evidence‐base in peri‐operative bleeding and transfusion, future work should focus on the rapid integration of the findings of clinical trials and clinical guidelines into clinical practice and reducing unwarranted variation in practice.

## Supporting information


**Appendix S1.** List of other relevant clinical guidelines.
