# Supplementary material for: Association of Anaesthetists guidelines: the use of blood components and their alternatives
Source: Anaesthesia. 2025 Jan 9;80(4):425–47. doi: 10.1111/anae.16542 (PMC11885198; doi:10.1111/anae.16542)
Supplement: Supplementary file 1 — Appendix S1. List of other relevant clinical guidelines. [file ANAE-80-425-s001.docx]

**Appendix S1** List of other relevant clinical guidelines

*Peri-operative anaemia*

Centre for Peri-operative Care (CPOC) 2022 ([https://cpoc.org.uk/guidelines-resources-guidelines/anaemia-perioperative-pathway](https://protect.checkpoint.com/v2/___https://cpoc.org.uk/guidelines-resources-guidelines/anaemia-perioperative-pathway___.bXQtcHJvZC1jcC1ldXcyLTE6dW5pdmVyc2l0eWhvc3BpdGFsc291dGhhbXB0b246YzpvOjFlZDM4NGQ2ZDZiNDVlYmEwMDQyMzhiNTExNjk1Yzk3OjY6NzNjNjozYTUyNjhmYzQ3ODZkMzcwMzAyMDM0ZDQ2ZmNkZTI0NzZkZDI2ZmMzZDAwYWYyYzVhMzMxOGVmZTU2ODQxZDY1OnA6VA))

Recommendations From the International Consensus Conference on Anemia Management in Surgical Patients (ICCAMS) 2023 ([https://doi.org/10.1097%2FSLA.0000000000005721](https://protect.checkpoint.com/v2/___https://doi.org/10.1097*2FSLA.0000000000005721___.bXQtcHJvZC1jcC1ldXcyLTE6dW5pdmVyc2l0eWhvc3BpdGFsc291dGhhbXB0b246YzpvOjFlZDM4NGQ2ZDZiNDVlYmEwMDQyMzhiNTExNjk1Yzk3OjY6YTBhMzpkNTBjMTBjZmVkM2FmMGYzZjgxOGJkZmFlYTQyYzIwMzc4MTE2MWZiZTYwMGI4NjI0YWM0ZjI1ZmE0YzhkNzRlOnA6VA))

UK guidelines on the management of iron deficiency in pregnancy 2020 – ([https://onlinelibrary.wiley.com/doi/full/10.1111/bjh.16221?sid=nlm%3Apubmed](https://protect.checkpoint.com/v2/___https://onlinelibrary.wiley.com/doi/full/10.1111/bjh.16221?sid=nlm%3Apubmed___.bXQtcHJvZC1jcC1ldXcyLTE6dW5pdmVyc2l0eWhvc3BpdGFsc291dGhhbXB0b246YzpvOjFlZDM4NGQ2ZDZiNDVlYmEwMDQyMzhiNTExNjk1Yzk3OjY6ZjFiOTozYWYyN2U0MDI1NjFjYzg0ZGFjNjJmN2EyNDVjZDRkZjhiMzhhMjg5OTRmZmNkYzEyZTM3NmUyYjI1NGEwY2U0OnA6VA))

*Patient blood management / general*

National Institute for Health and Care Excellence (NICE) 2015 – ([http://www.nice.org.uk/guidance/indevelopment/gid-CGWAVE0663](https://protect.checkpoint.com/v2/___http://www.nice.org.uk/guidance/indevelopment/gid-CGWAVE0663___.bXQtcHJvZC1jcC1ldXcyLTE6dW5pdmVyc2l0eWhvc3BpdGFsc291dGhhbXB0b246YzpvOjFlZDM4NGQ2ZDZiNDVlYmEwMDQyMzhiNTExNjk1Yzk3OjY6NzRiNjphNjAwYzgwMWZmZTY3OWNhYmE3ZGIxODkwNGU5ZDliNjI3MmNjNGRmOTdiNTI0M2I1OGJkOTU3NjBjY2I3OTRjOnA6VA))

Recommendations From the 2018 Frankfurt Consensus Conference 2018 – ([https://jamanetwork.com/journals/jama/fullarticle/2727453](https://protect.checkpoint.com/v2/___https://jamanetwork.com/journals/jama/fullarticle/2727453___.bXQtcHJvZC1jcC1ldXcyLTE6dW5pdmVyc2l0eWhvc3BpdGFsc291dGhhbXB0b246YzpvOjFlZDM4NGQ2ZDZiNDVlYmEwMDQyMzhiNTExNjk1Yzk3OjY6OGY0Njo4MjE0OGQ0MmViNjlmOTZlOWUxNzc5Nzk1NWU1ZWVjNDQ0YzdlZjhlMzdmMWNmODI2YjU2MzZiZDk4ZmFmOTBmOnA6VA))

National Blood Authority Australia PBM Guidelines ([http://www.blood.gov.au/pbm-guidelines](https://protect.checkpoint.com/v2/___http://www.blood.gov.au/pbm-guidelines___.bXQtcHJvZC1jcC1ldXcyLTE6dW5pdmVyc2l0eWhvc3BpdGFsc291dGhhbXB0b246YzpvOjFlZDM4NGQ2ZDZiNDVlYmEwMDQyMzhiNTExNjk1Yzk3OjY6ZjBlODozYjllYjhjYzg5YzI4YzY2ZGNhOWU4ZDgwNWM2ZmMzM2VmMzU0NWQ0OTNhZGUzZjQyM2FmOTRmNzVkNDg2YTdlOnA6VA))

Association of Anaesthetists guidelines: cell salvage for peri-operative blood conservation 2018 – ([https://associationofanaesthetists-publications.onlinelibrary.wiley.com/doi/10.1111/anae.14331](https://protect.checkpoint.com/v2/___https://associationofanaesthetists-publications.onlinelibrary.wiley.com/doi/10.1111/anae.14331___.bXQtcHJvZC1jcC1ldXcyLTE6dW5pdmVyc2l0eWhvc3BpdGFsc291dGhhbXB0b246YzpvOjFlZDM4NGQ2ZDZiNDVlYmEwMDQyMzhiNTExNjk1Yzk3OjY6MjYyZDo3MjRmZWY4MDczN2M2MWFjNjQzMWZjZWQyNjg4YTNkMWVlMjcxNjM1NGFkMTlhNDhlNDY0OTJjMmY4ZjhjMGZlOnA6VA))

*Major bleeding (trauma, cardiac and non-cardiac)*

British Society for Haematology Guideline: Haematological management of major haemorrhage 2022 – ([https://onlinelibrary.wiley.com/doi/10.1111/bjh.18275](https://protect.checkpoint.com/v2/___https://onlinelibrary.wiley.com/doi/10.1111/bjh.18275___.bXQtcHJvZC1jcC1ldXcyLTE6dW5pdmVyc2l0eWhvc3BpdGFsc291dGhhbXB0b246YzpvOjFlZDM4NGQ2ZDZiNDVlYmEwMDQyMzhiNTExNjk1Yzk3OjY6MjYzMjplNmY4MmE4ZDNkMGE1ZWZkNGQ4ZTQ3OWU4MjNiMjI4ZWUyM2VkYWZjYThlOTQ1NGNkYmNlNTIxMTZiY2YwYzlkOnA6VA))

Management of severe peri-operative bleeding: Guidelines from the European Society of Anaesthesiology and Intensive Care: Second update 2022 – ([https://journals.lww.com/ejanaesthesiology/fulltext/2023/04000/management_of_severe_peri_operative_bleeding_.2.aspx](https://protect.checkpoint.com/v2/___https://journals.lww.com/ejanaesthesiology/fulltext/2023/04000/management_of_severe_peri_operative_bleeding_.2.aspx___.bXQtcHJvZC1jcC1ldXcyLTE6dW5pdmVyc2l0eWhvc3BpdGFsc291dGhhbXB0b246YzpvOjFlZDM4NGQ2ZDZiNDVlYmEwMDQyMzhiNTExNjk1Yzk3OjY6YjM1NTpiOGZkMTE2MzhkZDczMmNlZGFiNTAwZDVjMDUzODFjODdmZDVlMmUwNzY1NTQ0YTExYTM4ODExNTRhNzJlMWM5OnA6VA))

Southampton Oxford Retrieval Team, Paediatric Major Haemorrhage – ([https://www.sort.nhs.uk/Media/Guidelines/Haemorrhage-quick-reference-guide.pdf](https://protect.checkpoint.com/v2/___https://www.sort.nhs.uk/Media/Guidelines/Haemorrhage-quick-reference-guide.pdf___.bXQtcHJvZC1jcC1ldXcyLTE6dW5pdmVyc2l0eWhvc3BpdGFsc291dGhhbXB0b246YzpvOjFlZDM4NGQ2ZDZiNDVlYmEwMDQyMzhiNTExNjk1Yzk3OjY6MjVlZjpmY2FkMDI2ZjNiNjlhMjM3ZGQ1ODI5YTE2MjkyN2ZlYzZlYzhkYzAzOWIzN2MxNjUyNTQ5NTVmNTZiYTgxY2ZhOnA6VA))

*Critical care*

Both from European Society of Intensive Care Medicine

Transfusion strategies in non-bleeding critically ill adults 2020 - ([https://link.springer.com/article/10.1007/s00134-019-05884-8](https://protect.checkpoint.com/v2/___https://link.springer.com/article/10.1007/s00134-019-05884-8___.bXQtcHJvZC1jcC1ldXcyLTE6dW5pdmVyc2l0eWhvc3BpdGFsc291dGhhbXB0b246YzpvOjFlZDM4NGQ2ZDZiNDVlYmEwMDQyMzhiNTExNjk1Yzk3OjY6MDNiMzpmODM2ODY5OWRkNGFiNWM4NDg1Mjc1M2M0N2VkYWFkNGViZTk2NGIxOTg3YTI2ZDQ2YjRkYjI3ZjA3ZDdmZmZkOnA6VA))

Transfusion strategies in bleeding critically ill adults 2021 – ([https://link.springer.com/article/10.1007/s00134-021-06531-x](https://protect.checkpoint.com/v2/___https://link.springer.com/article/10.1007/s00134-021-06531-x___.bXQtcHJvZC1jcC1ldXcyLTE6dW5pdmVyc2l0eWhvc3BpdGFsc291dGhhbXB0b246YzpvOjFlZDM4NGQ2ZDZiNDVlYmEwMDQyMzhiNTExNjk1Yzk3OjY6NGY5NTo5MjliYzQ4ODdmNmNiNTZkZjdjNzY5MjYzMDliZGEzNDYzN2JkYWNjZDhjOTk2YTgxZWRjODQ1NjFhMzBhMmQ0OnA6VA))

*Obstetrics*

Patient blood management in obstetrics: prevention and treatment of postpartum haemorrhage. A NATA consensus statement 2019 – ([https://www.bloodtransfusion.it/bt/article/view/243](https://protect.checkpoint.com/v2/___https://www.bloodtransfusion.it/bt/article/view/243___.bXQtcHJvZC1jcC1ldXcyLTE6dW5pdmVyc2l0eWhvc3BpdGFsc291dGhhbXB0b246YzpvOjFlZDM4NGQ2ZDZiNDVlYmEwMDQyMzhiNTExNjk1Yzk3OjY6Zjc2NjpmYzg1MzQ2ODI5MjI5NjBkYjA4NTRlMWIxOTIyMDdlNDBiY2VjYmMwZWMxNjYzNjQ2OGYzYjVhOGY4ZTFmYWY0OnA6VA))

Prevention and Management of Postpartum Haemorrhage (Green-top Guideline No. 52), Royal College of Obstetricians and Gynaecologists 2016 – ([https://www.rcog.org.uk/guidance/browse-all-guidance/green-top-guidelines/prevention-and-management-of-postpartum-haemorrhage-green-top-guideline-no-52/](https://protect.checkpoint.com/v2/___https://www.rcog.org.uk/guidance/browse-all-guidance/green-top-guidelines/prevention-and-management-of-postpartum-haemorrhage-green-top-guideline-no-52/___.bXQtcHJvZC1jcC1ldXcyLTE6dW5pdmVyc2l0eWhvc3BpdGFsc291dGhhbXB0b246YzpvOjFlZDM4NGQ2ZDZiNDVlYmEwMDQyMzhiNTExNjk1Yzk3OjY6MDQxZTplODJmYjZkYWUwMzE4ZTM0ZmQxNGI4OTkwMmY4N2UzMzE1ZTc5M2ViNjYxNGQ3YzVkMDg3MjY1ODU5M2VjYTYxOnA6VA))
